# Proximal femoral derotation osteotomy for management of femoral malversion: a systematic review

**DOI:** 10.1093/jhps/hnad024

**Published:** 2023-08-22

**Authors:** Mark Sohatee, Monketh Jaibaji, Ajay Malviya

**Affiliations:** The Royal Orthopaedic Hospital NHS Foundation Trust, Bristol North Road, Northfield, Birmingham B31 2AP, UK; Health Education England North East, Waterfront 4 Goldcrest Way, Newburn, Riverside, Newcastle upon Tyne NE15 8NY, UK; Department of Trauma and Orthopaedics, Northumbria Healthcare NHS Foundation Trust, Woodhorn Lane, Ashington, Northumberland NE63 9JJ, UK

## Abstract

Femoral malversion is an under-recognized contributor to hip pain in younger adults. Under treatment is often a contributor to poor outcomes in hip preservation surgery. We reviewed the literature to analyse the outcomes of proximal femoral derotation osteotomy as a treatment for femoral malversion as well as propose our own management algorithm for treating such patients.

A systematic review was conducted in accordance with Preferred Reporting Items for Systematic Reviews and Meta-Analyses guidelines searching four databases (PubMed, CINALH, MEDLINE and EMBASE) for studies investigating the outcomes of derotation osteotomy in treating malversion. Nine studies were found encompassing 229 hips.

At a mean follow-up of 39.9 months across the studies, there were only two conversions (1%) to total hip arthroplasty and four revision cases in total. Seven of the nine studies reported improved functional outcomes in their cohorts, with the mean Harris hip score improved from 63.7 to 87.3 where reported.

There is a paucity of literature around the outcomes of proximal femoral derotation osteotomy. However, both the evidence available and the authors’ experience suggest that consideration of femoral malversion is an essential component of hip preservation surgery, improving functional outcomes in cases of excessive femoral anteversion and femoral retroversion.

## INTRODUCTION

Femoral version (FV) is defined as the angular differences between the axis of the femoral neck and transcondylar axis of the knee; normal version in adults ranges from 10 to 15° [1]. If unrecognized, femoral malversion can contribute to poor outcomes in hip preservation surgery [2–4]. Further difficulty arises as the measurement varies according to the imaging modality used [5, 6].

Femoral malversion is a common contributor to hip and groin pain [7], and version abnormalities are postulated to be a cause of impingement and instability around the hip as well as contribute to patella maltracking and patellofemoral arthritis [5, 8–10]. Any association with degenerative changes around the hip is poorly defined. Femoral version is a key determinant of hip range of motion [11, 12]. Increased FV is associated with decreased external rotation and extension of the hip; this may lead to ischiofemoral impingement presenting with deep gluteal pain [13]. The obligatory internal rotation needed to initiate gait is responsible for the intoeing often seen in children, particularly those with neuromuscular disorders such as cerebral palsy; where derotational osteotomy is utilized to correct [14]. Other biomechanical abnormalities include a shortened abductor lever arm of the abductors causing hip instability [1], and anterior hip pain may occur due to psoas irritation. Anterior knee pain and patellofemoral instability can also occur with increased external rotation due to patella maltracking, and there is an increased association with anterior cruciate ligament rupture [15]. Conversely, femoral retroversion is associated with external rotation of the hip and decreased internal rotation. There is an association with labral and articular cartilage due to increased impingement of the femoral neck on the acetabulum predisposing to osteoarthritis [16]. Abnormal FV can also be present in other predefined hip pathologies such as developmental hip dysplasia (DDH) or femoroacetabular impingement [17], and the relative contribution to the patient’s symptoms can be challenging to elucidate. There is no clear evidence on when to address malversion in this context.

Despite recent developments understanding, the literature regarding treatment of femoral malversion is sparse. Expectations in this cohort of patients will differ from that of the young adult population where a much higher functional outcome is required, and tolerance of failed correction will be lower. There is also limited evidence to support a particular surgical technique.

The primary purpose of the review is to provide an overview of the current evidence for proximal femoral derotation osteotomy in treating abnormal FV. Based on the available literature and the senior author’s experience, we aimed to suggest a viable management algorithm for such cases.

## MATERIALS AND METHODS

A systematic review was conducted in accordance with the Preferred Reporting Items for Systematic Reviews and Meta-Analyses (PRISMA guidelines). We conducted a search of four databases (CINALH, EMBASE, MEDLINE and PUBMED) for English language articles using the following search terms ‘Proximal femur OR femur’ AND ‘Osteotomy’ AND ‘Derotation’ OR ‘De-rotational’ OR ‘Rotational’, published up to July 2022. The following inclusion criteria were agreed upon (i) studies investigating the outcomes of derotation osteotomies in the subtrochanteric region of the femur used to treat abnormalities of FV, (ii) studies reporting clinical outcomes assessing hip function and (iii) skeletally mature patients.

We judged skeletally mature patients to be above the age of 12 years as the triradiate cartilage fuses at this age [18]. Patients with cerebral palsy were excluded. Case reports and conference proceedings were excluded.

As the paucity of data available in the published literature was part of the rationale for conducting the review, we decided not to include a cut-off for methodological quality of the studies or follow-up time.

Two authors independently ran the search and reviewed the studies against the inclusion criteria. The literature search returned 1318 results. Following the removal of duplicates, 671 results remained. Following screening of the abstracts, 33 studies were selected for full-text analysis. Eight studies were selected for inclusion in the review by this process. The references of these studies were checked to see if any other studies met the inclusion criteria. Two studies were found by this method leading to a total of 10 studies.

**Fig. 1.**
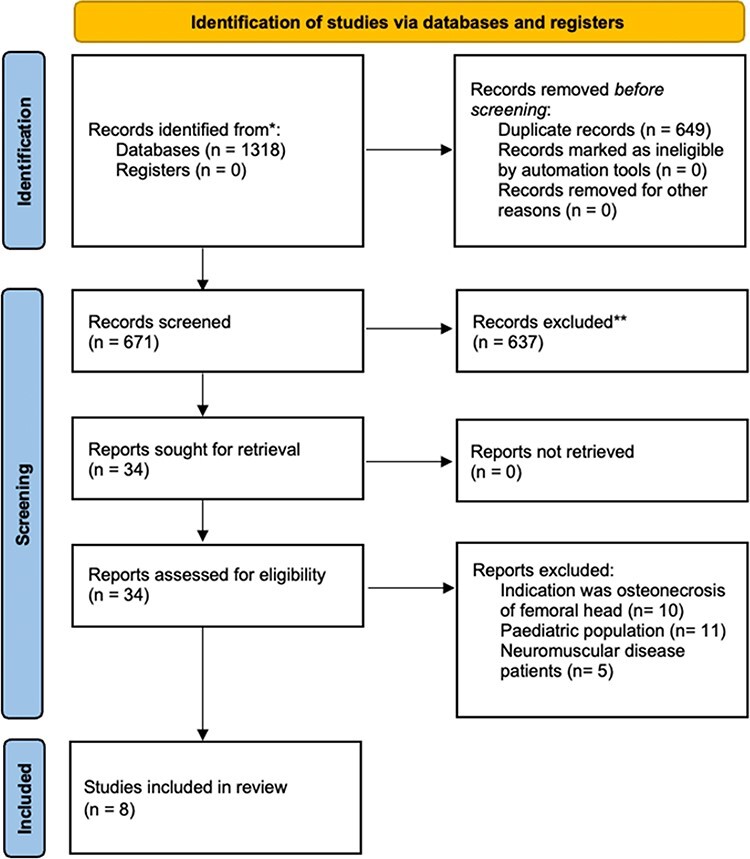
PRISMA flow diagram.

Two studies [19, 20] were published from the same institution and had some overlap in their cohorts, but reported different outcomes. A consensus among the authors was reached to exclude the smaller study from any analysis. Full details of the search process and reasons for exclusions are shown in the PRISMA flow diagram in Fig. 1.

## STATISTICAL ANALYSIS

Statistical analysis on categorical data was performed using cross-tabulation and chi-squared testing for categorical data or Fisher’s exact test where sample size did not permit chi-squared testing. Between-group comparison of continuous data was performed using a *t*-test. Data were analysed using JMP 16.2. A *P* value of <0.05 was considered statistically significant.

Studies were independently assessed for methodological quality using the methodological index for non-randomized studies (MINORS) criteria [21]. Assessment was performed independently by the first two authors, and disagreement was resolved by discussion with the remaining authors with final arbitration going to the senior author.

## RESULTS

### Study characteristics

The literature search identified 10 eligible manuscripts; however, only eight had data presented in a way that could be included in the analysis, and one of these studies was excluded from statistically analysis due to a cohort overlap [19] (Table I). Within these seven papers, there were 206 cases in total that had sufficient data presented for statistical analysis. (Table II). Most cases (52%) were derotation osteotomies conducted for excessive FV.

**Table I.** Summary of studies

*Author*	*Year*	*Study type*	*Evidence level*	*Number of hips*	*Mean age*	*Mean F/u (months)*	*Femoral torsion*	*Retroversion*	*Tibial torsion*	*Male–female*	*Technique (i.e. nail, plate)*	*Average correction*	*Time to FWB (weeks)*	*Non-union*
Huber *et al.* [47]	2009	Retrospective case series	IV	22^a^	15	NR	0	22	NR	10:4	LCP	NR	NR	0
Pailhé *et al.* [22]	2014	Retrospective case series	IV	9	13.6	23	9	0	NR	2:4	IM nail	19 ± 4º	10.4	0
Buly *et al.* [24]	2018	Retrospective case series	IV	55	29	78	39	16	NR	7:36	IM nail	24º in retroversion, 23º in anteversion	NR	1
Kamath *et al.* [23]	2015	Retrospective case series	IV	28	21.4	NR	26	2	NR	05:21	Plate	NR	6	1
Rigling*et al.* [25]	2020	Retrospective case series	IV	25	24	37	7	18	NR	10:15	LCP	Not stated	8	0
Hatem *et al.* [26]	2021	Retrospective case series	IV	37	33	24	15	22	NR	8:26	IM nail	NS	6	2
Lerch *et al.* [20]^b^	2022	Retrospective case series	IV	25	26	48	25	0	36 ± 11º	1:24	DCP	20 ± 3º	NR	0
Lerch *et al.* [19]^b^	2022	Retrospective case series	IV	20	29	12	14	6	39 ± 10 (increasing FV) 30 ± 3 (decresed FV)	3:17	DCP	NR	NR	NR
Tönnis *et al.* [3]	1999	Retrospective case series	IV	7	NR	NR	NR	NR	NR	NR	DCP	NR	NR	NR
Mastel *et al.* [27]	2021	Retrospective case series	IV	29	29	19.8	0	29	NR	NR	IM nail	NR	0	2

Abbreviations: NR, not recorded.

a Study had three further skeletally immature patients included.

b Overlapping cohorts.

**Table II.** Overall outcomes across six studies (where data could be analysed)

*Variable*	*Number*
Total hips	237
Total cases of increased femoral anteversion (where reported)	120/229 (42%)
Total cases of femoral retroversion (where reported)	109/229(48%)
Total hips eligible for analysis	177
Mean age	28 (range 12–59)
Mean FU months	33 (range 7–65)
Mean time to FWB (weeks)	8
Mean of preoperative HHSs (where recorded)	63.7
Mean of postoperative HHSs	87.3

Abbreviations: FU, follow-up.

Three of six studies encompassing 69 hips provided data of the presenting symptoms. 33/69 patients reported lumbar pain, 25 reported ipsilateral knee pain, eight reported inguinal pain and five reported pains in the trochanteric region. Patients in the study by Paihlé et al [22] all reported frequent tripping during athletic activities and pain around the hip or knee. Kamath *et al.* [23] reported that patients experienced improvements in hip and trochanteric pain postoperatively; however, they did not specify the preoperative symptoms.

The mean age of patients included within the study was 28 years (range 12–59). The mean follow-up for all manuscripts was 33.3 months (range 7–65), and within all the studies, the mean time to allowing full weightbearing (FWB) was 6 weeks (range 0–10 weeks).

### Outcomes

Three studies utilized the Harris hip score (HHS) [24–26]. The mean score improved from 63.7 preoperatively to 87.3 postoperatively (Table II) at a mean follow-up of 40 months. Rigling *et al.* [25] demonstrated an improvement in Western Ontario and McMasters index score from 3 to 1 (*P* < 0.01). Two studies used the subjective hip value [20, 25] with a reported improvement from 31 preoperatively to 79 postoperatively (*P* < 0.01) at a mean follow-up of 29 months across the two studies. Hatem *et al.* [26] reported improvement in mean Oswestry disability index from 45% ± 16 to 22% ± 17 (*P* = 0.001). Lerch *et al.* [19] reported improved subjective hip values (20 ± 22 to 81 ± 11) and mean Merle d’Aubigné–Postel score (14 ± 1 preoperatively to 17 ± 1 at final follow-up).

### Concurrent osteotomies

Only Buly *et al.* [24] reported performing tibial derotation osteotomies to correct compensatory increased tibia torsion. Lerch *et al.* [20] did report two patients in their series with ongoing knee pain at final follow-up due to ongoing tibial torsion (20° and 30°, respectively). Nine periacetabular osteotomies (PAOs) were performed across four studies [19, 20, 23, 24]. One case in the series by Lerch *et al.* [20]was conducted due to severe acetabular retroversion. PAOs were performed in three patients in the study by Buly *et al.* [24] due to severe dysplasia. The indications were not stated in the remaining six cases, which were from the series by Kamath *et al.* [23] and Lerch *et al.* [19].

Only one study performed any objective gait analysis [19]. Lerch *et al.* [19] analysed 20 patients undergoing derotation osteotomy, 14 for increased FV and six for decreased FV. In the 14 cases of increased FV, the mean foot progression angle (FPA) increased from 1.3 ± 7° to 4.5 ± 6°. Preoperatively, 5/14 had an intoeing gait compared to 2/14 at the final follow-up of 12 months. In their six patients with reduced FV, the mean FPA reduced from 8.2 ± 8° to 0.5 ± 5°. In addition, the incidence of out-toeing gait reduced from 2/6 to 0/6 at final follow-up. This study [19] also reported significantly improved clinical outcomes; however, these were not analysed due to the overlapping cohorts with the larger study published by Lerch *et al.* [20]. Pailhé *et al.* [22] reported all abnormalities related to intoeing gait corrected postoperatively in their series. Rigling *et al.* [25] reported one case of asymptomatic intoeing in their postoperative outcomes.

### Methods of fixation

Six studies described the use of a locking compression plate (LCP) or dynamic compression plate (DCP), and four of the studies described the use of an intramedullary (IM) nail (Table V).

### Complications

Complications were discussed in 7/10 studies (Tables III, IV and V), with four cases of revision surgery being required, two conversions to total hip arthroplasty (THA), one infection, one repeat osteotomy due to lack of correction and 58 instances (32.9%) where metalware removal was required. Lerch *et al.* [20] reported that three of their 25 cases required subsequent hip arthroscopy for adhesiolysis. Mastel *et al.* [27] had three instances of intraoperative fracture, all of which were fixed with the addition of a cerclage wire followed by a period and protective weightbearing; they all went on to unite. They had three cases of delayed union, all of which eventually united without the need for revision surgery.

**Table III.** Complications identified

*Author*	*Complications*
Huber *et al.* [47]	Not reported
Pailhé *et al.* [22]	1 revision for varus displacement, 2 cases of distal locking screw removal
Kamath *et al.* [23]	1 revision for implant failure
Buly *et al.* [24]	2 conversions to Total hip arthroplasty mean of 30.5 months, 1 further osteotomy for further correction and 1 late infection, 30 cases of implant removal
Rigling *et al.* [25]	6 cases of hardware removal required (due to metalware irritation), 1 case of over correction (5º retroversion to 39º anteversion), 1 case of under correction (47º anteversion to 39º)
Hatem *et al.* [26]	1 repeat osteotomy due to the lack of improvement after initial surgery, 1 case of delayed union requiring locking screw removal 6 months postoperatively, 4 cases of asymptomatic grade 1 HO
Lerch *et al.* [20]	3 cases of further hip arthroscopy for adhesiolysis, 16 cases of metalware removal
Tönnis *et al.* [3]	Not reported
Mastel *et al.* [27]	3 cases of intraoperative fracture, 2 cases of non-union

Abbreviations: HO, heterotopic ossification.

Note: The study by Lerch *et al.* [19] was excluded from analysis, and no complications were reported in this study.

**Table IV.** Analysis of complications

		*Complications requiring reoperation/further operation*				
*Author*	*Method of fixation*	*Revision/further surgery required*	*Conversion to THA*	*Infection*	*Delayed/non-union*	*Over/under correction*
Huber *et al.* [47]	LCP	No complications stated				
Pailhé *et al.* [22]	IM nail	1				
Kamath^a^ *et al.* [23]	LCP	1				
Buly *et al.* [24]	IM nail	1	2	1		
Rigling *et al.* [25]	LCP					1
Hatem *et al.* [26]	IM nail	1			1	
Lerch *et al.* [20]	DCP	3	0	0	0	0
Tönnis *et al.* [3]	Not reported	No complications stated				
Mastel *et al.* [27]	IM nail	2			2	

Note: The study by Lerch *et al.* [19] was excluded from analysis.

a Excluded from analysis.

Subanalysis of complications based on the method of fixation (Intramedullary nailing versus LCP/DCP) was undertaken (Table IV).

Complications were seen in 12/129 (9%) versus 4/78 (5%) where a fixation construct utilizing a plate was implemented. This was not statistically significant (*P* = 0.6).

Considering the need for metalware removal (Table V), there were 47/129 (36%) in (IM) nailing versus 22/78 (28%) where a fixation construct utilizing a plate was implemented. This was not statistically significant (*P* = 0.27).

**Table V.** Metalware removal

*Author*	*Fixation*	*Metalware removal (%)*
Huber *et al.* [47]	LCP	NR
Pailhé *et al.* [22]	IM nail	2 (27)
Kamath* *et al.* [23]	LCP	0 (0)
Buly *et al.* [24]	IM nail	30 (46)
Rigling *et al.* [25]	LCP	6 (22)
Hatem *et al.* [26]	IM nail	4 (10)
Lerch *et al.* [20]	DCP	16 (64)
Tönnis *et al.* [3]	DCP	NR
Mastel *et al.* [27]	IM nail	11 (27)
Overall		37%

Note: The study by Lerch *et al.* [19] was excluded from analysis.

Abbreviations: NR, not recorded.

### Investigations

All studies utilized the range of motion testing and plain radiographs as first-line investigations. CT scan was the modality of choice in all studies to quantify the degree of rotational deformity. Lerch *et al.* [20], Rigling *et al.* [25] and Buly *et al.* [24] utilized magnetic resonance (MR) arthrograms. Mastel *et al.* [27] utilized MR arthrograms in all patients to look for intra-articular pathology. A CT was performed in cases where the initial MR did not include a ‘femoral torsional protocol’. Only two studies [19, 22] included a gait analysis as part of their standard pre- and postoperative assessment. The study by Rigling *et al.* [25] was the only study of MR arthrogram postoperatively to assess correction.

### Quality assessment

The mean MINORS score was 11 (range 6–14). Breakdown of the MINORS scores is shown in Table VI. Common reasons for low scores included the retrospective nature of the studies and low patient numbers as well as short follow-up.

**Table VI.** MINORS scores

*Study*	*Q1*	*Q2*	*Q3*	*Q4*	*Q5*	*Q6*	*Q7*	*Q8*	*Q9*	*Q10*	*Q11*	*Q12*	*Total*
Huber *et al.* [47]	2	2	0	0	0	0	2	0	0	0	0	0	6
Pailhé *et al.* [22]	2	2	0	2	0	2	2	0	0	0	0	0	8
Kamath *et al.* [23]	2	0	0	2	0	2	2	0	0	0	0	2	10
Buly *et al.* [24]	2	0	0	2	0	2	2	0	0	2	2	2	14
Rigling *et al.* [25]	2	2	0	2	0	2	0	0	0	2	2	2	14
Hatem *et al.* [26]	2	2	0	2	0	2	2	0	0	0	0	2	12
Lerch *et al.* [20]	2	2	0	2	2	2	2	0	0	0	2	2	14
Lerch *et al.* [19]	2	2	0	2	2	2	2	0	0	0	2	2	14
Tönnis *et al.* [3]	2	2	0	0	0	0	2	0	0	0	0	0	6
Mastel *et al.* [27]	2	2		2	2	2	2	0	0	0	2	2	14

## DISCUSSION

This review has demonstrated that outcomes of proximal femoral derotation osteotomy are under-reported in the literature. Across the 178 hips suitable for analysis, there was a significant improvement in HHS at a mean follow-up of 39.9 months. All studies that assessed pateint reported outcome measures (PROMS) reported statistically significant improvements in postoperative scores.

**Fig. 2.**
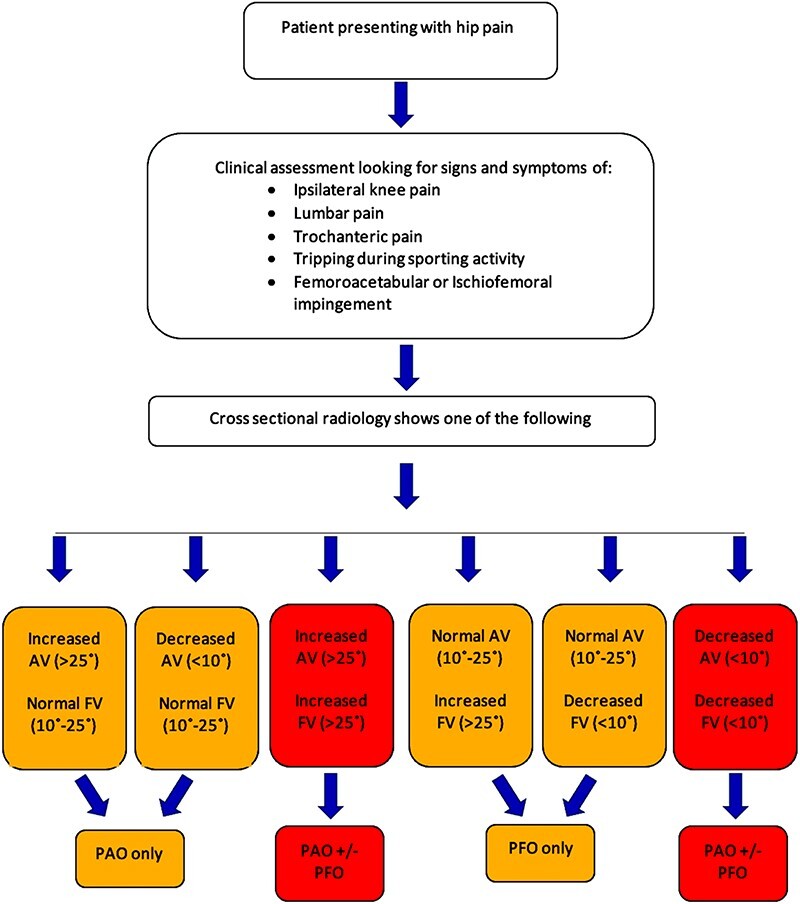
Algorithm for the workup and management of hip pain in a young adult. Abbreviations: PFO, proximal femur osteotomy, PAO, periacetabular osteotomy, FV, Femoral version.

Derotational femoral osteotomies are often combined with other surgeries such as hip arthroscopies, tibial derotation osteotomies and PAOs. In the study by Kamath *et al.* [23], these included labral repair (eight), relative head neck lengthening (16), PAO (four) and shortening femoral osteotomy (11). In the study by Lerch et al. [20], 15/20 patients had an additional Cam resection performed at the time of procedure with two of their cohort subsequently undergoing PAO [19]. Excessive tibial torsion, which is often present in increased FV, can be exaggerated by derotation osteotomy [28]. This can lead to the patient having an increased external foot progression angle (FPA), which can precipitate other lower extremity problems [29]. Increased FV can be challenging to identify in cases of compensatory tibial torsion as an intoeing gait will typically not be present as previously reported by Lerch *et al.* [30]. A subsequent study by the same group demonstrated a reduced FPA in their preoperative cohort, with normal FPA being defined as 0–15° [19]. Derotational osteotomies to correct increased FV resulted in an increase in FPA across their whole cohort of 15 patients from 1.3 ± 6.6° to 4.5 ± 5.9°. The FPA at final follow-up is similar to that of their normal control group. Only 5/15 patients with increased FV and visible intoeing reduced to two at final follow-up. They demonstrated markedly improved patient-reported outcome measures and high patient satisfaction. This study, though limited by low numbers and being retrospective in nature, is the clearest evidence to date of the use of gait analysis to recognize the presence of posterior extra-articular impingement to increase FV. However, the nature of preoperative pathology present, along with the concomitant procedures performed to correct it, will significantly affect patient outcomes. This makes the effect of derotation osteotomy in isolation difficult to study. This review does, however, demonstrate the role PFO that plays in wider deformity correction, Fig. 2. In addition, the union rates are reliably high and complication rates are low, with metalware irritation being the only consistent problem; however, we suspect this may be due to the studies having a low threshold for metalware removal, especially in younger patients.

Reports in the literature regarding thresholds for normal FV have differed. Tönnis *et al.* described grades of increased and decreased acetabular version (AV) and FV, relative to the assumed normal range [3]. In their description, normal version is defined as 10–25°, with 21–25° being ‘moderately’ increased version and >25° being ‘severely’ increased version. Retroversion is defined as 10–14° and ‘severe’ retroversion is <10° according to their classification [3]. Lerch *et al.* [17] define severely increased version as >35° with severe retroversion as <0°. Other literature has reported <5° as the threshold to define femoral retroversion [6, 31, 32], and >22° has also been reported as the threshold for femoral anteversion [33]. There is no clear consensus regarding the threshold for normal values. Different methods of measurement exist using different imaging modalities, and these have reported a variation in values as will be discussed later. This requires careful consideration in patients with FV measured between 25–35° and 5–10°, as to correction of version should be attempted [34]. In our institution, we have selected 10° as the cut-off for retroversion and >30° for excessive anteversion.

Most of the osteotomies included were to correct excessive femoral anteversion (60%), with the remaining cases being performed for retroversion (decreased FV). These pathologies have a varying impact on patients’ functional outcomes and symptoms. Increased FV is associated with decreased abductor power due to reduced femoral offset and associated hip instability. Psoas irritation has also been linked, possibly due to the tendon acting as a dynamic hip stabilizer because of this reduced offset [35]. Posterior trochanteric impingement can also impact mobility. There has been an increased awareness in the published literature of the association between excessive version and osteoarthritis of the hip, which is likely due to excessive shear forces at the periphery of the acetabulum during ambulation, accelerating the degeneration of articular cartilage [36]. This problem can be compounded in the presence of DDH, which is why the authors recommend screening using CT for version abnormalities of the femur in all cases of DDH and conducting a derotation osteotomy in addition to PAO. PAO alone in these patients may not be sufficient for addressing the biomechanical abnormalities responsible for their symptoms.

Femoral retroversion can also lead to femoroacetabular impingement; however, this occurs anteriorly between femoral neck and acetabulum. This can also precipitate labral tears and cartilage damage, leading to osteoarthritis. It has been postulated that untreated retroversion may be a factor in failed arthroscopic management of hip impingement [16], and retroversion is also strongly linked to slipped upper femoral epiphysis [37]. Therefore, planning of osteotomies should consider segmental variation to ensure healthy hip biomechanics and prevent further clinical problems [1].

Although these pathologies originate from the same source and both are linked to hip pain and reduced range of motion, the biomechanical features do differ. Due to low numbers and the heterogeneity of the studies, it was difficult to conduct subgroup analysis between patients with increased and decreased FV. Both abnormalities are also associated with lumbar spine pain owing to transfer of stress secondary to limited hip range of motion. The impact around the knee is also worth considering . Intoeing gait, caused by excessive anteversion can cause patella maltracking and increases knee adduction moments. Both can lead to patellofemoral osteoarthritis as well as instability. Although the outcomes in treating knee patellofemoral abnormalities is beyond the scope of the review, it is still important to note good outcomes in derotation osteotomies in improving patellofemoral pain [38, 39].

Although presenting features were not accurately documented in 50% of the studies in this review, it is implied from the manuscripts that hip pain was the most common complaint. Femoral malversion is increasingly being recognized as a contributing pathology in patients with hip pain attributed to Femoroacetabular impingement (FAI). In a cross-sectional study, Lerch *et al.* examined the prevalence of malversion in 538 hips with 11 predefined hip pathologies [17]. They noted that 52% of patients had abnormal FV (>25° or <10°). One in six of their cohort had, what they defined as a severe malversion (>35° or <0°). Their study showed an association with severe anteversion and dysplasia, but perhaps more interestingly, both increased FV and decreased FV were seen across all defined pathologies. Nineteen patients included in the study had no predefined diagnosis but presented with hip pain; 74% of these patients were found to have at least moderate malversion of the femur. The findings within their cohort study justify consideration of FV in any algorithm in managing young adult hip pathology. The concern of the authors is that derotation osteotomy is under-utilized as an option in these cases. Despite the findings of Lerch *et al.* [17], earlier studies such as that by Tibor *et al.* [40] have been less conclusive about the associations between malversion and symptomatic pathology. Their study was smaller in size (112 patients) and found no association between femoral malversion and markers of dysplasia (lateral centre edge angle or alpha angle). However, they utilized two-dimensional correlations that may have been skewed by patients with normal values. An issue more broadly present in the literature is the heterogeneity of measurement techniques, and imaging modalities used may also lead to an under-recognition of malversion.

The role of imaging in assessing malversion remains undefined. Tönnis *et al.* [3] have described techniques for judging version on plain radiographs; however, cross-sectional imaging is far more accurate in quantifying the degree of abnormality. The studies reported seemed to favour clinical examination followed by CT scanning. Botser *et al.* [33] compared the version angles obtained using CT and MRI scanning as well as correlating with clinical examination. While both imaging modalities should have good inter-rater correlation, absolute values obtained on CT scan were significantly higher than those obtained on MRI. Therefore, patients could be included or excluded based on which imaging modality is used. Moreover, while there was a strong correlation between the rotational profile of the hip and the version angle measured on both imaging modalities, there was still an overlap between the retroversion, normal version and excessive anteversion groups, suggesting that diagnosis should not be dependent on one investigative technique. This also serves to make patient selection for derotation osteotomy challenging.

Lerch *et al.* [17] expanded on the potential clinical scenarios that could arise from femoral malversion, in conjunction with acetabular malversion and related this to the McKibbin instability index. This gives rise to nine possible subgroups with normal version of both, two instances where the McKibbin index will be compensated, four instances where there will have moderately abnormal McKibbin index and two instances of ‘aggravated’ McKibbin index. Although the treatment for these nine subgroups is yet to be defined, it stands to reason that treatment should be focused on those who have a moderate abnormality and aggravated McKibbin index with appropriate symptomology. Patients with acetabular dysplasia and/or disorders of AV may need a concurrent acetabular reorientation procedure such as a PAO [41].

The need to address intra-articular pathology is another important consideration, Mastel *et al.* [42] a yewar following their initial cohort study included in the review published a matched cohort study comparing patients undergoing derotation osteotomy with patients who only underwent hip arthroscopy. Both groups had similar outcomes; however, they identified one patient in the hip arthroscopy group who was dissatisfied following a resection of a CAM lesion and had good outcomes following a subsequent derotation osteotomy. They further identified three patients in their derotation osteotomy group who had ongoing symptoms despite multiple arthroscopies. This demonstrates the importance of addressing the underlying mechanical pathology. However, whether the intra-articular pathology needs to be address following derotation osteotomy is less clear. It may be, for example, that a large CAM lesion is asymptomatic in the context of normal FV, while a small CAM lesion may be symptomatic in the case of malversion.

Based on the available evidence and the authors’ experience, we propose the management algorithm in Fig. 2. The approach recognizes both FV and AV as key factors in FAI [41]. Both ultimately have a significant influence on the hip range of motion especially internal and external rotation [41]. Increased FV combined with AV results in a high McKibbin index, which increases hip internal rotation and may contribute to anterior instability. The combination of low FV and a low AV led to a low McKibbin index and can aggravate anterior impingement. Combined abnormalities are also associated with hip pain in the absence of FAI [41, 43]. Gollwitzer *et al.* [44] recently published a treatment algorithm for the management of ischiofemoral impingement (IFG). Following symptomatic treatment of IFG, they propose a targeted injection of the quadriceps femoris muscle as both the pain relieving and diagnostic intervention to judge the cause of the patients’ symptoms. Their management algorithm then goes on to discuss treatment of underlying pathology, which does included increased femoral anteversion. Our algorithm does highlight symptomatic IFG as a presenting feature of femoral anteversion and would recommend targeted CT-guided infiltration of the quadratus femoris muscle in cases of clinical uncertainty. The decision to operate should be based on the presence of relevant symptoms in conjunction with a demonstrable radiological abnormality. The authors recommend CT scan as the primary mode of investigation due to its utility operative planning and perceived greater sensitivity in detecting malversion [33]. MRI is utilized in cases where there are concerns that additional soft tissue pathology may be contributing to the patients’ symptoms.

When comparing the two surgical techniques, there were no statistically significant differences in complications. However, IM nailing does offer theoretical advantages of reduced soft tissue stripping and shorter scar length, and hence, it is the senior author’s preferred technique. As the osteotomy site does not need to be exposed in IM nailing, there is improved preservation of the periosteal blood supply; however, the findings of this study do not demonstrate these impacts on the clinical outcome. IM nailing also allows for earlier weightbearing. A disadvantage of IM nailing is the potential disruption of the hip abductors while accessing the femoral canal, although none of the three studies that used IM nailing reported this to be an issue. This is particularly significant in the case of increased FV as the abductors will already have a shortened lever arm [45]. The plate does allow for direct compression at the osteotomy site, which theoretically promotes bony union [23, 30]. Ultimately, both devices appear safe to use with a similar complication and reoperation profile.

The study by Rigling *et al.* [25] was the only study to report revision osteotomy for overcorrection. The correction technique varies for both methods of fixation. While all studies used a predefined correction based on preoperative imaging, plate fixation utilizes parallel k wires proximal and distal to the osteotomy site secured with a Verbrugge clamp. Studies using the IM nailing technique either utilize a sterile protractor and rotation of the limb, which is in traction, or use a Steinmann pin with a guide to rotate the distal fragment. It may be that more sophisticated guides used in the IM nailing reduce the risk of overcorrection. It should also be noted that in the study by Rigling *et al.* [25], the patient who underwent revision for overcorrection did still perceive benefit from the original surgery.

Concerns have been raised of the unintended consequences of derotation osteotomies on coronal plane alignment. Nelitz *et al.* [46] conducted a study using 3D modelling, which concluded that inter-trochanteric derotation osteotomy increased varus angulation of the hip, while conducting the osteotomy in the supracondylar level increased valgus angulation of the femur. The level of the osteotomy will also impact muscle attachments, which may influence gait [46]. The clinical relevance of this remains unproven but does illustrate a need for careful preoperative planning required before undertaking such procedures.

Further clinical studies are needed to define the optimal level of the osteotomy and analyse the clinical relevance of changes in 3D alignment as well as whether this can be addressed by modifications to the surgical technique. It may be that success is related to individual surgeon proficiency in that hip surgeons prefer to perform the osteotomy in the subtrochanteric region and knee surgeons may prefer performing the osteotomy in the supracondylar region.

While available results are encouraging, they must be considered in the context of the limitations in the quality of the studies. First, no prospective or randomized studies were included. Low numbers and lack of control also limited the quality of the statistical analysis that could be conducted from the data. The low numbers also meant that the subgroup analysis was limited, and finally, the heterogeneity in reporting outcomes meant that a meta-analysis of PROM outcomes was not feasible. This is a common problem when conducting systematic reviews.

## CONCLUSION

Awareness of torsional abnormalities of the femur is paramount in effective joint preservation surgery of the hip. Subtrochanteric derotation osteotomy can be performed in addition to other corrective procedures such as PAO and can be supplemented with hip arthroscopy. Our literature search has revealed a paucity of literature investigating PFO in young adults without neuromuscular conditions. It is unclear whether PFO is underused in deformity correction or its outcomes are simply under-reported. Further studies to evaluate the outcomes of derotation osteotomies with longer follow-up and the evaluation of PROMs are required. A further study is planned by the authors looking at long-term outcomes in patients treated using our proposed algorithm.

## Data Availability

This statement is to confirm that we hold all the raw data for this systematic review.

## WHAT IS KNOWN ABOUT THE SUBJECT

The role of femoral malversion in young adults with hip pain is slowly being recognized; however, the literature is sparse when it comes to the reporting of the outcomes of proximal femoral osteotomy(PFO) for malversion.

## WHAT THIS STUDY ADDS

This is the largest review of the outcomes of PFO to correct malversion. The outcomes are integrated with the literature search and the authors’ own experience to create a management algorithm for patients with version abnormalities of the hip.
